# The acute metabolic responses to Fastin-RR^®^, a commercial weight loss/energy product, in overweight and obese individuals

**DOI:** 10.1186/1550-2783-10-S1-P11

**Published:** 2013-12-06

**Authors:** Patrick L Jacobs

**Affiliations:** 1 Superior Performance Research, Miami FL 33186, USA

## Background

While over the counter weight loss products have grown into one the largest categories of nutritional supplements, most advertising claims for these products are limited to proven effects of individual ingredients and generally demonstrated in fit, active college aged males. Few commercial weight loss products have been properly examined in finished commercial form and seldom have been studied in the overweight and obese populations. The purpose of this study was to investigate the acute metabolic effects of the commercial weight loss/energy product, Fastin-RR^®^ (High-Tech Pharmaceuticals, Inc., Norcross, GA) in overweight and obese men and women.

## Methods

Eleven men (n=6) and women (n=5), 28.5 ± 5 years of age with BMI between 25 and 35, voluntarily participated in this research study. All research participants completed three 6-hour resting metabolic testing sessions in which three treatment conditions were examined in randomized order including Fastin-RR^R^ (FAS), 300 mg caffeine anhydrous (CAF), and cellulose placebo condition (PL). Metabolic activity was determined in 15 minute intervals at baseline and 45 minutes, 1½ hr, 3hrs, 4½ hrs and 6 hrs following ingestion. Metabolic activity was determined with open flow spirometry (VO2000, Medgraphics, St. Paul, MN) with outcomes including oxygen consumption (VO_2_), respiratory exchange ratio (RER), minute ventilation (V_E_) and oxygen extraction (VO_2_/V_E_). Values of metabolic variables were adjusted into change scores relative to baseline levels. Statistical analyses were conducted using a 3x6 ANOVA (condition X time) for repeated measures with the accepted level of significance set at p<0.05.

## Results

Analyses revealed no significant differences between conditions at baseline in values of VO_2_, V_E_, or RER. Results indicated that VO_2_ change scores for FAS were significantly greater at all time points following ingestion (+22.1%, +18.9%, +15.9%, +12.6%, +8.4%) compared with PL (0.4%, -1.7%, -2.3%, -1.1%, 0.5%) and compared with CAF ( +6.3%, +6.5%, +7.1%, +4.2 %, +3.6%) (p’s < 0.05). Similar response patterns were observed for V_E_ as VO_2_ with FAS: (+26.6, +22.9%, +23.3%, +18.7%, +9.0%), CAF (+6.3%, +9.4%, +7.8%, +7.6%, +9.3%) and PL (-1.3%, -2.5%, -1.9%, -3.6%, +3.1%). The FAS V_E_ change scores were significantly greater than CAF and PL at 45 min, 90min and 3 hrs (p<0.05). The RER change scores with PL and CAF were within 2% of baseline values across the six hours of testing. In contrast, FAS produced a pattern of declining values of RER over time to 9% and 11% below baseline at 4½ hrs and 6 hrs post ingestion, respectively, which were significantly less than CAF and PL.

## Conclusion

These findings indicate that resting energy expenditure is significantly enhanced with Fastin-RR^®^.

There was approximately 16.6% increase in resting energy expenditure over the first three hours of testing with an increase of over 14.5% in energy uptake over the entire six hour period. These findings also indicate that Fastin-RR^®^ produced a substantial shift in energy substrate utilization with significantly greater levels of fat oxidation.

## Funding

This study was supported by funding from Hi-Tech Pharmaceuticals, Inc., Norcross, GA, USA.

